# Probing the Use of Homemade Carbon Fiber Microsensor for Quantifying Caffeine in Soft Beverages

**DOI:** 10.3390/ma16051928

**Published:** 2023-02-25

**Authors:** Karla Caroline de Freitas Araújo, Emily Cintia Tossi de Araújo Costa, Danyelle Medeiros de Araújo, Elisama V. Santos, Carlos A. Martínez-Huitle, Pollyana Souza Castro

**Affiliations:** 1 Institute of Chemistry, Federal University of Rio Grande do Norte, Av. Campus Universitário, Av. Salgado Filho 3000, Lagoa Nova, Natal CEP59078-970, RN, Brazil; 2 National Institute for Alternative Technologies of Detection, Toxicological Evaluation and Removal of Micropollutants and Radioactives (INCT-DATREM), Institute of Chemistry, Universidade Estadual Paulista, Araraquara CEP14800-900, SP, Brazil; 3 School of Science and Technology, Federal University of Rio Grande do Norte, Av. Campus Universitário, Av. Salgado Filho 3000, Lagoa Nova, Natal CEP59078-970, RN, Brazil

**Keywords:** caffeine, carbon fiber, microelectrode, cyclic voltammetry, beverages

## Abstract

In the development of electrochemical sensors, carbon micro-structured or micro-materials have been widely used as supports/modifiers to improve the performance of bare electrodes. In the case of carbon fibers (CFs), these carbonaceous materials have received extensive attention and their use has been proposed in a variety of fields. However, to the best of our knowledge, no attempts for electroanalytical determination of caffeine with CF microelectrode (µE) have been reported in the literature. Therefore, a homemade CF-µE was fabricated, characterized, and used to determine caffeine in soft beverage samples. From the electrochemical characterization of the CF-µE in K_3_Fe(CN)_6_ 10 mmol L^−1^ plus KCl 100 mmol L^−1^, a radius of about 6 µm was estimated, registering a sigmoidal voltammetric profile that distinguishes a µE indicating that the mass-transport conditions were improved. Voltammetric analysis of the electrochemical response of caffeine at the CF-µE clearly showed that no effects were attained due to the mass transport in solution. Differential pulse voltammetric analysis using the CF-µE was able to determine the detection sensitivity, concentration range (0.3 to 4.5 µmol L^−1^), limit of detection (0.13 μmol L^−1^) and linear relationship (*I* (µA) = (11.6 ± 0.09) × 10^−3^ [caffeine, μmol L^−1^] − (0.37 ± 0.24) × 10^−3^), aiming at the quantification applicability in concentration quality-control for the beverages industry. When the homemade CF-µE was used to quantify the caffeine concentration in the soft beverage samples, the values obtained were satisfactory in comparison with the concentrations reported in the literature. Additionally, the concentrations were analytically determined by high-performance liquid chromatography (HPLC). These results show that these electrodes may be an alternative to the development of new and portable reliable analytical tools at low cost with high efficiency.

## 1. Introduction

Caffeine or 1-3-7-trimethylxantine is a white crystalline xanthine alkaloid that promotes various effects on the body’s metabolism when ingested, including stimulating the central nervous system, increasing blood pressure in the short term, and secreting gastric acid. This drug is widely used in different concentrations in coffee, cola nuts, cocoa beans, tea leaves, cola beverages and several pharmaceutical substances used worldwide [1,2,3]. Therefore, the concentration of caffeine in its various origins should be controlled.

The most common technique used for analyzing/quantifying this bioactive compound is high-performance liquid chromatography (HPLC) [3,4,5,6,7]. However, this instrumental method involves high-cost analysis, is time-consuming, and requires calibration as well as maintenance as compared with other approaches. Among the competitive methods with HPLC, electrochemical methodologies have received great attention in the last years due to their advantages, allowing significant technical impacts in the form of easy-to-upscale, versatility, effectiveness, cost-effective balance, easy-to-automatize, and the development of small portable devices [8,9,10,11,12]. These technological features are mainly dependent on the sensor materials used because these materials (commonly named electrodes) may be adapted by size and/or different materials characteristics [13]. Therefore, the versatility of electrode materials, in terms of size and nature, has been widely investigated depending on the concentration control area (e.g., food, pharmaceutical and beverages industries) visioning to reach a single-step reagentless analysis with high detection efficiency by using a smaller active electrode area [2,14,15,16,17,18,19].

Concerning the electroanalytical detection of caffeine, various sensing-materials have been examined and reported in the existing literature; however, these electrode materials are expensive, and their preparation methodologies are dependent on time-consuming procedures and modification steps sensor [20,21,22,23]. In this sense, more sensing-elaboration strategies have been investigated for improvements, in sensibility, selectivity, and detection reliability, using traditional materials; for example, the size of the electrochemical sensor [24,25,26,27]. Consequently, the miniaturization is a hot-spot investigation topic because it has allowed us to better understand the chemical/electrochemical processes that take place on the micro- and nano- environments as well as provide significant new benefits as real-time monitoring and high sensitivity as a consequence of the high and efficient mass transport [25,28,29].

In the development of electrochemical microsensors, carbon materials have been widely used as supports due to their simplicity and active surface to improve the performances in specific concentration control areas [30,31]. Among the various types of carbon materials, carbon fibers have found extensive use in a variety of fields, including supercapacitors, sensors, biomedical applications, etc., because of their desirable properties. Shape, high chemical stability, high strength, high electrical conductivity, high surface area, outstanding electrocatalytic activity, and compatibility with matrix materials are just a few of its characteristics. Therefore, CF-µE has recently attracted interest due to their remarkable qualities [31,32].

Based on the existing literature, only few reports have described the applicability of carbon-based sensors to determine caffeine in food, beverages, drugs, and medications. However, to the best of our knowledge, no attempts for electroanalytical determination of caffeine with CF-µE have been reported in the literature. Therefore, a homemade CF-µE was fabricated, characterized, and used to determine caffeine, estimating the concentration linear range, calibration function, and determination of caffeine in soft beverage samples.

## 2. Experimental Methods

### 2.1. Reagents

All reagents were of analytical grade, and all aqueous solutions were prepared using a high-purity water obtained from a Millipore Milli-Q system with resistivity >18 MΩ at 25 °C. H_2_SO_4_ and caffeine (purity 98%) were purchased from Quimex and Isofar, respectively. K₃[Fe(CN)₆] and KCl were purchased from Synth (São Paulo, Brazil). Solutions were daily prepared under constant agitation for 30 min before each experiment.

### 2.2. Homemade CF-µE Fabrication

**Figure 1:**
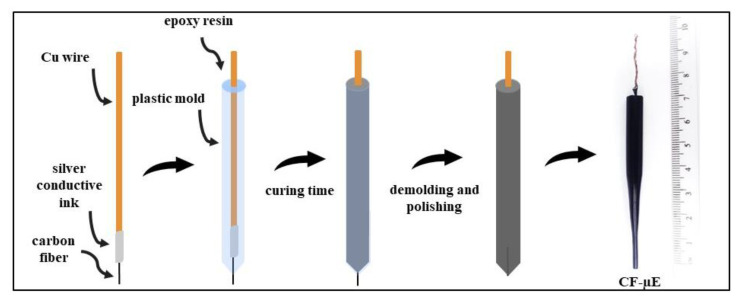
Scheme for the CF-µE homemade fabrication.

A cleaned 3 cm length carbon fiber was connected to a Cu wire (⌀ 1 mm diameter and 12 cm length) with silver conductive ink (Joint Metal Comércio LTDA (SP) Brazil). After drying, this set was carefully inserted into a plastic mold, filled with epoxy resin SQ 2119-PT (Avipol (SP), Brazil), and held in an upright position. For curing and demolding, the 48-hour time was respected. Then, the CF-µE was polished using 1200 and 1500 grit sandpaper until obtaining a smooth and flat surface containing a carbon fiber microdisk. Finally, the microelectrode was washed thoroughly with distilled water, air-dried, inspected by using an optical microscope (Olympus BX51M, Thermo Fisher Scientific, Waltham, MA, USA) to prevent any defects and stored away from dust. A scheme for the CF-µE fabrication is provided in the Figure 1.

### 2.3. Electrochemical Measurements

Voltammetric analysis (cyclic voltammetry (CV) and differential pulse voltammetry (DPV)) were performed with an Autolab PGSTAT302 by using a three-electrode cell consisting of an Ag/AgCl (3 mol L^−1^ KCl) as a reference electrode, a platinum wire as a counter electrode, and a homemade CF-µE as the working electrode. The CF-µE radius was estimated recording a CV in K_3_Fe(CN)_6_ 10 mmol L^−1^ plus KCl 100 mmol L^−1^ solution. The caffeine electroanalytical experiments were carried out at 25 °C and five CVs were recorded for each measurement in the potential range from +0.5 to +1.7 V with scan rate of 50 mV s^−1^ in 0.5 mol L^−1^ H_2_SO_4_ as the supporting electrolyte in the presence and absence of caffeine 0.1 mol L^−1^. The effect of the scan rate was also studied at 20, 40, 60, 80, and 100 mV s^−1^ in the presence of caffeine in 0.5 mol L^−1^ H_2_SO_4_. The DPV parameters to quantify caffeine, using 0.5 mol L^−1^ H_2_SO_4_ as a supporting electrolyte, were equilibration time = 10 s, initial potential = +0.5 V, final potential = +1.7 V, potential scan rate = 50 mV s^−1^, pulse amplitude = +0.05 V, and modulation time: 0.04 s. The above-optimized parameters were used for all measurements. Then, the calibrations curves (peak intensity vs. caffeine concentration in the range from 0 to 6 µmol L^−1^) were studied by least-square linear regression, and the obtained figures (slopes and intercepts) were reported with their confidence interval, *p* = 95%. Reproducibility and stability parameters were also evaluated. Homemade CF-µE was cleaned recording ten CV cycles from +0.60 V to +1.80 V at 100 mV s^−1^ in 0.5 mol L^−1^ H_2_SO_4_ before each one of the measurements. Caffeine determinations with CF-µE were validated by a reverse-phase HPLC (Shimadzu LC-6 Series, Berlin, Germany) equipped with a Nucleosil C18 column, Berlin, Germany (4.6 × 250 mm), and an UV–vis detector set at 273 nm. An acetonitrile/water mixture (25:75 % *v*/*v*) was used as the mobile phase at a flow rate of 0.6 mL min^−1^, injecting 20 μL of each sample. The retention time (t_r_) was 6.8 min.

### 2.4. Electrochemical Determination of Caffeine in Beverages

Evaluating the practical feasibility of the homemade CF-µE, the caffeine concentration in soft beverage samples was determined. Ultrasonication (10 min) was used to eliminate the gas from commercial soft drinks, which were then transferred to the electrochemical cell with the supporting electrolyte to proceed with the caffeine detection (0.5 mL to 4.5 mL of supporting electrolyte). The standard addition method was used to quantify caffeine in the samples to minimize the possible matrix effects due to the presence of other components in the real samples. All experiments were carried out in triplicate, and mean values (standard deviation < 5%) were used for the figures.

## 3. Results and Discussion

### 3.1. Fabrication and Characterization of the CF-µE

**Figure 2:**
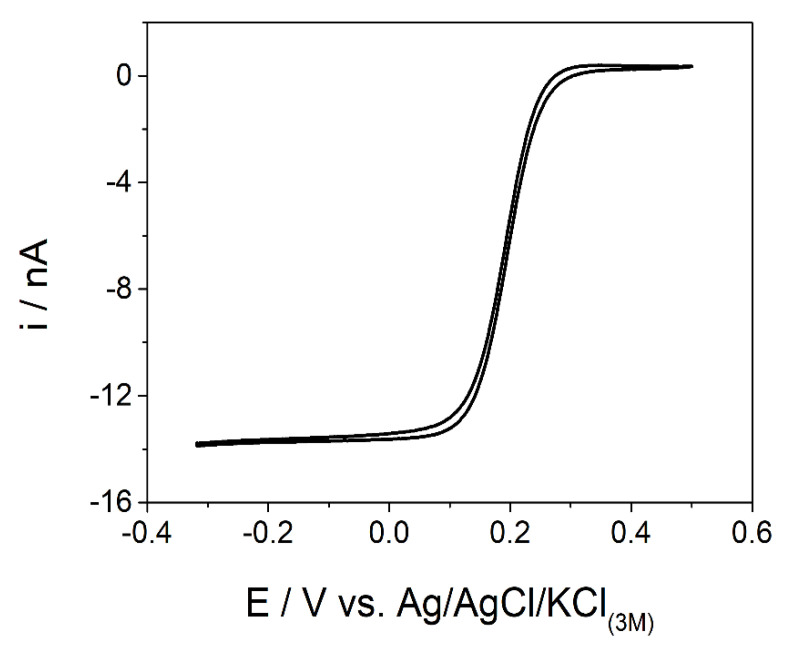
Cyclic voltammogram recorded with the CF-µE using K_3_[Fe(CN)_6_] 10 mmol L^−1^ in KCl 100 mmol L^−1^ solution; ν = 30 mV s^−1^; r = 6 µm.

Prior to evaluating the analytical performance of the CF-µE as a sensor for caffeine, it was necessary to inspect the electrodic surface by using an optical microscope and isolate the area. This procedure is extremely important to ensure the quality of the analytical results by using a flat and defect-free surface containing only a carbon fiber microdisk. Then, the CF-µE was characterized by CV using a K_3_Fe(CN)_6_ 10 mmol L^−1^ plus KCl 100 mmol L^−1^ solution. In Figure 2, a steady-state response owing to the enhanced mass-transport conditions was obtained giving a sigmoidal shape, characteristic of microelectrodes. By using the Equation (1), the radius of the CF-µE was found to be 6 µm.

*I*_L_ = 4nF*D*C*r* (1)

where *I*_L_ is the limiting current at the steady-state condition (A), n is the number of electrons involved on the electrodic reaction, F is the Faraday constant (96,485 C mol^−1^), *D* is the diffusion coefficient (cm^2^ s^−1^), C is the bulk concentration of the electroactive species (mol cm^−3^), and *r* is the radius of the microdisk electrode (cm) [33].

### 3.2. Cyclic Voltammetry Experiments in Presence of Caffeine

**Figure 3:**
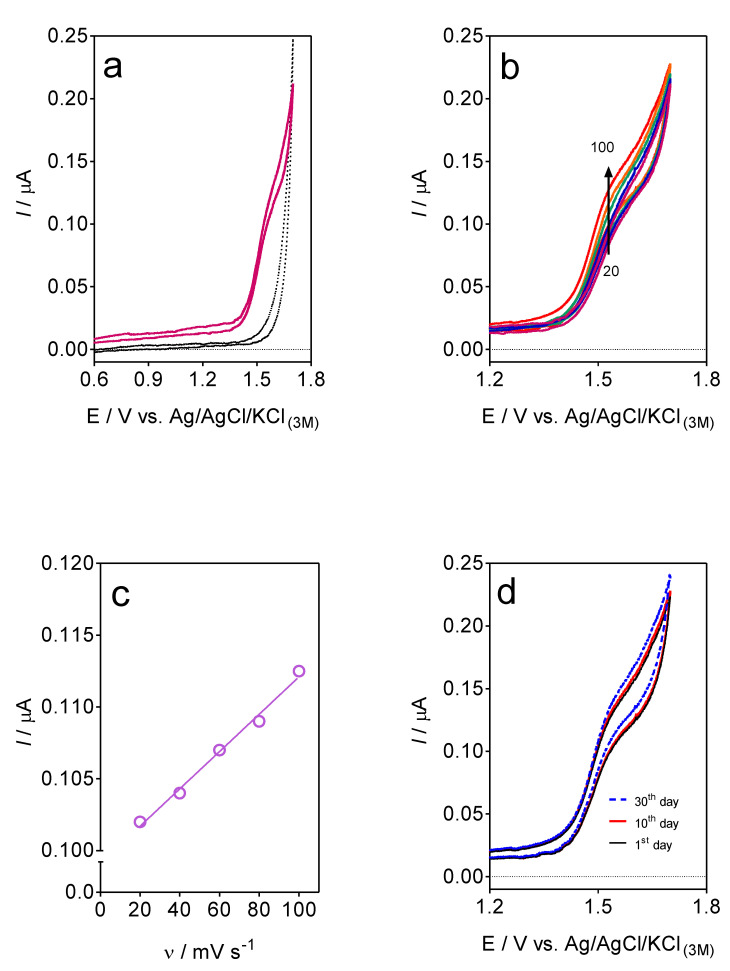
(**a**) CVs recorded at CF-µE in 25 mL of 0.5 mol L^−1^ H_2_SO_4_ solution (black curve) and 100 µL of 0.1 mol L^−1^ caffeine in 25 mL of 0.5 mol L^−1^ H_2_SO_4_ solution (red curve), scan rate: 10 mV s^−1^; (**b**) scan rate effect (20 (pink line), 40 (blue line), 60 (green line), 80 (orange line) and 100 mV s^−1^ (red line)) as a function of the electrochemical response of caffeine using 100 µL of 0.1 mol L^−1^ caffeine in 25 mL of 0.5 mol L^−1^ H_2_SO_4_; (**c**) oxidation peak current (*I_pa_*) values versus the scan rates (*v*), *I_pa_* (µA) = 1.3 × 10^−4^ (µA mV s^−1^) + 0.098, r^2^ = 0.9928; (**d**) CVs recorded at CF-µE in 100 µL of 0.1 mol L^−1^ caffeine in 25 mL of 0.5 mol L^−1^ H_2_SO_4_ solution for the first, tenth, and after 30 days; r = 6 µm.

**Figure 4:**
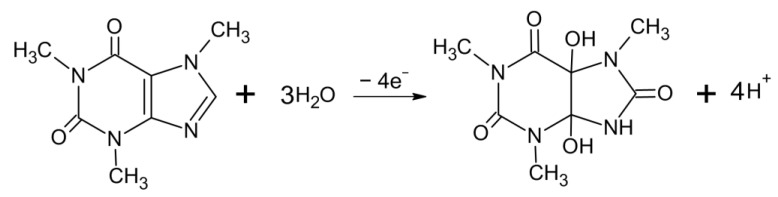
Mechanism of overall oxidation of caffeine.

As a preliminary result, the electrochemical response for the electroactive species in solution (supporting electrolyte and caffeine) was investigated by using the CV technique (Figure 3). In the supporting electrolyte (Figure 3a), the CF-µE did not exhibit significant current responses. Conversely, a clear voltammetric oxidation response was registered at +1.5 V in the presence of caffeine in solution (Figure 3a), while no cathodic response was observed on the reverse scan, indicating that the oxidation is an irreversible process [14,27,34,35]. This voltammetric behavior is in agreement with the results reported elsewhere where the caffeine oxidation mechanism is a 4e^−^, 4H^+^ process. Following the mechanism described in the existing literature, oxidation byproducts such as uric acid and its diol-analog are formed and, subsequently, these intermediates are rapidly fragmented [2,36] (see scheme in Figure 4).

The voltammetric effect of the scan rate (20–100 mV s^−1^) as a function of the electrochemical response of caffeine was also investigated using 100 µL of 0.1 mol L^−1^ caffeine in 25 mL of 0.5 mol L^−1^ H_2_SO_4_. As observed in Figure 3b, the oxidation peak current (*I_pa_*) values at different scan rates showed a negligible difference. In addition, a slight shift on the oxidation potential (E*_pa_*) was attained to more positive potential values when the scan rate was increased. These results visibly showed that no effects were attained due to the mass transport in solution because the miniaturization of the electrode surface provides significant profits in the sensitivity.

The oxidation peak current (*I_pa_*) values versus the scan rates (*v*) are shown in Figure 3c, demonstrating a good fitting linear relationship between them (*I_pa_* (µA) = 1.3 × 10^−4^ (µA mV s^−1^) + 0.098), with a coefficient of determination over 0.9928. From these results, it was possible to confirm that the CF-µE has a higher mass transfer rate and reaction rate. This behavior is associated to the dimensions of the CF-µE that reduces its mass transfer rate of the caffeine oxidation, resulting in the amplification of the electrochemical response. In fact, the signal is significantly improved with respect to the result previously achieved when a macro-electrode was used [11], in terms of the voltammetric signal, concentration range, noise, and so on.

The reproducibility of the electrochemical signal of the CF-µE was tested by consecutive 10 cyclic potential scans in the presence of caffeine (100 µL of 0.1 mol L^−1^ caffeine in 25 mL of 0.5 mol L^−1^ H_2_SO_4_), at a scan rate of 100 mV s^−1^. The result clearly showed that no significant changes in the caffeine oxidation-current were observed after five voltammetric cycles, showing that the CF-µE response is stable. After that, the CF-µE was taken out from the solution, washed with deionized water, and exposed to the air for several days. Afterward, a similar voltammetric test was carried out again after 10 and 30 days [14,34]. As can be observed in Figure 3d, all of the CV curves are similar (the voltammetric profiles at 1st, 10th, and 30th day), in terms of the electrochemical current responses, indicating that this homemade microelectrode is exceptionally stable and has significant reproducibility, and consequently, it can be considered as a potential sensing-tool to quantify caffeine in the liquid samples.

### 3.3. Differential Pulse Voltammetric Experiments

**Figure 5:**
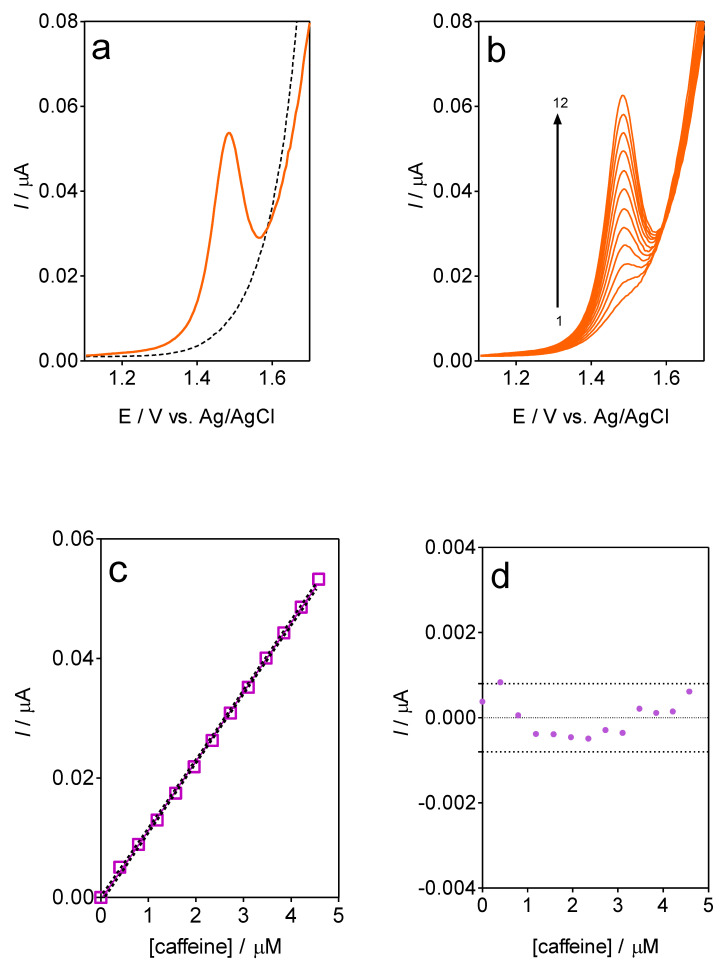
DPV profiles at CF-µE in 0.5 mol L^−1^ H_2_SO_4_ as supporting electrolyte (**a**) in absence (dashed line) or presence (orange full line) of caffeine in solution, (**b**) standard additions of caffeine solution (0.1 mol L^−1^): (1) 0.40, (2) 0.79, (3) 1.19, (4) 1.57, (5) 1.96, (6) 2.34, (7) 2.72, (8) 3.10, (9) 3.47, (10) 3.84, (11) 4.21, and (12) 4.58 µmol L^−1^. DPV parameters were of initial potential = 0.5 V; final potential = 1.8 V; potential scan rate = 10 mV s^−1^, pulse amplitude = 50 mV and slow agitation. (**c**) Linear calibration plot of caffeine concentration in solution versus current peak, based on the data collected from (**b**), using CF-µE in acidic (0.1 mol L^−1^ H_2_SO_4_) medium; r = 6 µm. (**d**) Graphic displays weighted residuals.

The detection sensitivity of the CF-µE was assessed by the DPV analysis (Figure 5), aiming the quantification applicability in concentration quality-control for the beverages industry. The relevant parameters of this experiment are reported in the Material and Methods section. The DPV signal for caffeine was registered at approximately +1.48 V (Figure 5a), which agrees with the current-signal recorded at the CV study (Figure 3). Then, a linear relationship between the peak current and the caffeine concentration for the CF-µE was obtained via the evaluation of different caffeine concentrations (0–6 µM) in 0.5 mol L^−1^ H_2_SO_4_. As can be seen in Figure 5b, an increase in the current-response was attained when the concentration of caffeine in the acidic solution was increased by, at least, twelve analyte concentration additions. This protocol was carried out in triplicate, and after that, the analytical curves were also constructed and compared.

In this sense, a linear relationship between 0.3 to 4.5 µmol L^−1^ (r^2^ = 0.9994) (inset in Figure 5c) was found by plotting the peak current intensity as a function of the caffeine concentration, obtaining *I* (µA) = (11.6 ± 0.09) × 10^−3^ [caffeine, μmol L^−1^] – (0.37 ± 0.24) × 10^−3^ (slope and intercept were the average of three independent calibrations). The limit of detection (LOD) was estimated from the equation LOD = 3.3 × *S*_y/x_/*b*, where *S_y/x_* is the residual standard deviation and *b* is the slope of the calibration plot, which was approximately 0.13 μmol L^−1^. Additionally, the residuals of the regression were randomly distributed around the zero (inset, Figure 5d), allowing a visual verification of the absence of a significant nonlinearity [27]. These approaches (LOD and residuals) are able to control both false positive and false negative errors (α = β = 0.05), as recommended by IUPAC [37,38] as well as already established by experts in the field [38,39]. It is important to indicate that additional calibration curves were obtained on different days in order to verify the good stability of the CF-µE by no registration of alterations in the statistical values (relative standard deviation (RSD), which were about 1.92% [38]). Then, the results clearly demonstrated that the CF-µE presented good repeatability and reproducibility in the analytical measurements performed, avoiding time-consuming procedures associated with the chemical cleaning or pre-treatment procedures for its surface.

Based on the existing literature, various electrochemical sensors have been constructed and used for quantifying caffeine in beverages. In this context, the proposed CF-µE has been compared with the other results (Table 1); however, even when the figures of merit are similar in some cases, the dimensions of our miniaturized sensor was able to obtain a higher sensitivity and selectivity. For example, the DPV responses achieved with other carbonaceous materials were nearly to 12.5 [1], 6.5 [40], and 3 µA [41] for the concentrations of about 0.4, 28 and 0.8 µM by using areas of approximately 28.3 mm^2^, 12.6 mm^2^, and 0.72 cm^2^, while that, in this work, a DPV signal of 0.038 µA was achieved for 0.79 µM with a reduced surface area of about 113.1 µm^2^. These insights evidence the advantages of our CF-µE over other sensors because it can be easily fabricated for sustainable practices in the spirit of green chemistry, avoiding high volumes of reagent wastes and enabling time- and cost-efficient research, achieving significant responses at lower caffeine concentrations. Another feature is that this CF-µE can be inexpensive because it was not mixed or modified with other materials, as illustrated in the examples in Table 1. Generally speaking, our CF-µE can be used in the caffeine quality-control concentration in beverages, but more investigations are needed for evaluating other additional experimental conditions, surface areas, and modifiers to significantly improve the LOD in order to find supplementary applications.

### 3.4. Caffeine Determination in Soft Beverage Samples

**Figure 6:**
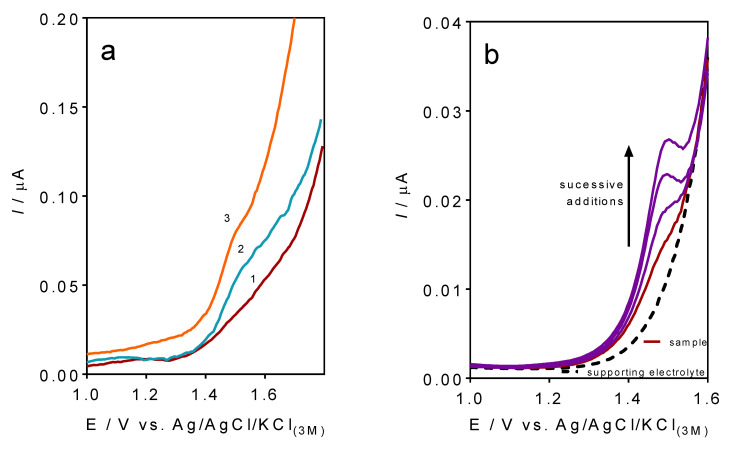
DPV analysis of (**a**) some drink samples ((1) caffeine-free soft drink, (2) cola–soft drink 1 and (3) cola–soft drink 2), and (**b**) the standard addition procedure for the cola–soft drink 2 (plot with the DPV profile of supporting electrolyte), the soft beverage sample as well as the 1° addition, 2° addition, and 3° addition of 0.1 mol L^−1^ caffeine, with CF-µE. DPV parameters were of initial potential = 0.5 V; final potential = 1.8 V; potential scan rate = 10 mV s^−1^, pulse amplitude = 50 mV.

CF-µE was used to control the caffeine concentration in several soft beverage samples by the standard addition protocol with the DPV analysis (Figure 6). This procedure was implemented in order to diminish the effects of other components in the soft beverage samples (matrix effect). Firstly, as indicated in the Experimental section, all beverage samples were ultrasonicated to remove the gas content. After that, laboratory samples were prepared by diluting 0.2 mL of each one of the samples under investigation with the supporting electrolyte (5 mL) to operate in the linear range of the method. On the one hand, a caffeine-free soft drink was also examined, aiming to test a possible contribution of the matrix itself to the analyte signal. On the other hand, cola beverages were also electrochemically evaluated. As shown in Figure 6a, no voltammetric signal around the caffeine potential was registered. Meanwhile, a current-voltammetric response was achieved at all beverages samples, confirming the presence of caffeine in the composition of these samples (see some examples in Figure 6a).

In the case of soft drinks containing caffeine, as described in the product information, three consecutive standard additions (100 µL of 0.1 M caffeine) were conducted in the electrochemical cell [40,55]. Figure 6b shows that the standard additions promoted an increase in the current-signal, also confirming the presence of the caffeine in the drink. Another important feature is that no significant alterations in the caffeine oxidation peak were achieved (Figure 6b), as observed on other sensors reported in the literature [9,14,40,41,54,55], which is significantly associated with the effects on mass transport that were minimized by the dimensions of the CF-µE. The known amount of caffeine added to the samples was able to estimate the recoveries.

The mean results were obtained for the standard additions protocol at each one of the soft drinks analyzed, recording three measurements with acceptable standard deviations and confidence intervals relating to a probability of 95%. This strategy consents to validate both false positives and false negatives (α = β = 0.05), as recommended by the IUPAC [37,39]. Subsequently, all samples were also analyzed by HPLC, and the results were compared with the DPV analysis of the CF-µE (Table 2). Analyzing the figures reached, CF-µE can be considered an efficient tool to be employed with good confidence in the caffeine concentration evaluation of soft beverage samples. The caffeine concentrations measured in the soft beverage samples with the CF-µE were like those quantified by HPLC (as an independent method with 95% of confidence [37,39]), and comparable to those reported in the nutritional table of the samples.

### 3.5. Interference Studies

**Figure 7:**
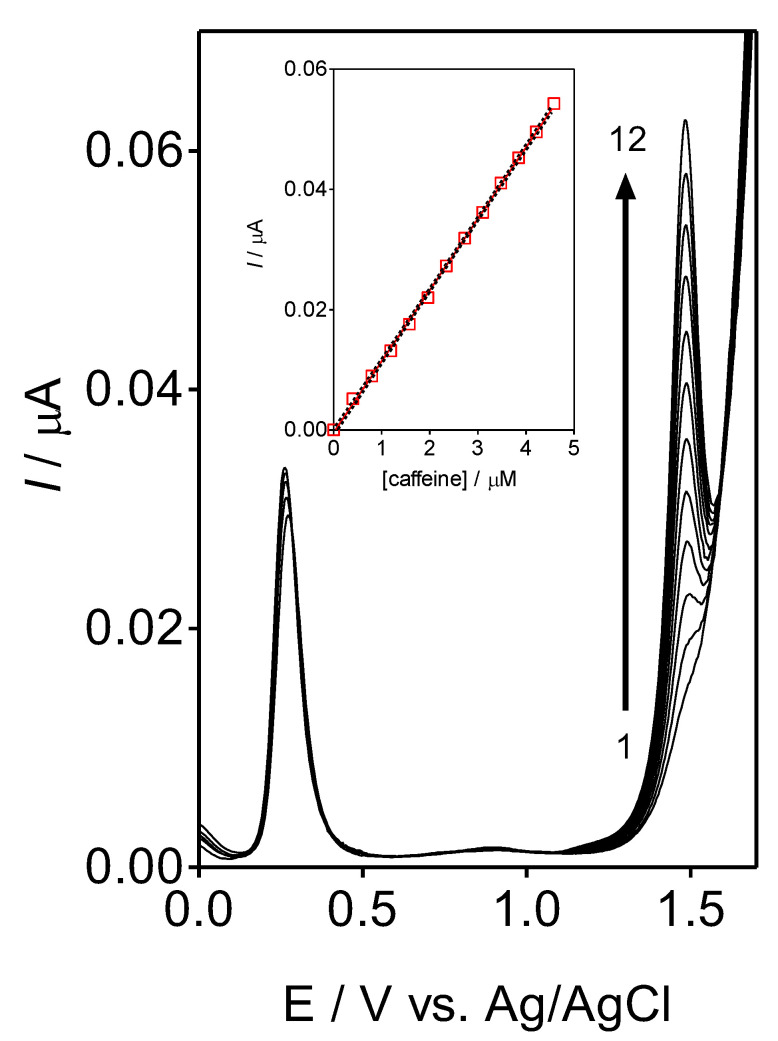
DPV profiles at CF-µE in 0.5 mol L^−1^ H_2_SO_4_ as supporting electrolyte for contructing the analytical curve of caffeine (standard additions of caffeine solution (0.1 mol L^−1^): (1) 0.40, (2) 0.79, (3) 1.19, (4) 1.57, (5) 1.96, (6) 2.34, (7) 2.72, (8) 3.10, (9) 3.47, (10) 3.84, (11) 4.21, and (12) 4.58 µ mol L^−1^) in presence of ascorbic acid (50 µmol L^−1^) in solution. Inset: Linear calibration plot of caffeine concentration in solution versus current peak, based on the data collected from the analytical curve.

As described, CF-µE presented good performance for quantifying caffeine. The selectivity of this sensor was also evaluated by intentionally introducing concentrations of ascorbic acid as interference during caffeine analysis. Ascorbic acid, also known as Vitamin C, is a water-soluble vitamin found in citrus and other fruits and vegetables; also, it is a supplement in the soft beverages. Therefore, this acid was chosen to test the determination of caffeine in the presence of an important interference. The experimental data are reported in Figure 7. The voltammetric signals for the ascorbic acid and caffeine were clearly registered at +0.05 V and + 0.4 V, respectively. The results showed that an excess of the concentration of ascorbic acid into the sample solution did not cause interference on the determination of caffeine, demonstrating the viability of this electrochemical sensor. In fact, no significant modifications were observed on the voltammetric responses of caffeine, in terms of electrical potential and current intensities, when a new analytical curve was obtained in the presence of ascorbic acid.

## 4. Conclusions

A homemade CF-µE was fabricated and it was a suitable electrochemical microsensor for caffeine determinations. Under the experimental conditions examined, caffeine displayed irreversible behavior in cyclic voltammetry with the CF-µE. This sensor exhibited a high detection sensitivity, a high mass transfer rate, and an enhanced signal-to-noise ratio; consequently, it is shown to have extraordinary stability and reproducibility, making it more applicable as a sensor in the food control analyses and measurements. For future works of this research field, some modifiers could be investigated in order to increase the surface area of the microsensor to guarantee more stability, sensibility, and selectivity for real-time monitoring.

## Figures and Tables

**Table 1:** Selected examples of quantification of caffeine in soft drink beverages at different electrochemical sensors. Comparison of LOD at different electrodes.

Electrodes	Sample	Method	Electrolyte	LOD/µmol L^−1^	LOD/ppm	Ref.
^1^ CA-ZnFe-modified GCE	Coffee and commercial beverages	DPV	1 mol L^−1^ H_2_SO_4_	10.0	0.194	[35]
^2^ CuS NPs MCPE	Commercial tea and coffee samples	DPV	Acetate buffer (pH 7.0)	0.018	0.00035	[18]
Pt@ZnCo_2_O_4_	Beverage and energy drink	Amperometric	0.1 mol L^−1^ H_2_SO_4_	0.05	0.000971	[42]
Nafion/GCE	Cola beverages	DPV	0.1 mol L^−1^ H_2_SO_4_	0.798	0.0155	[34]
Nafio-covered lead film electrode	Tea, coffee, soft and energy drink samples, and pharmaceutical formulation	DPV	0.1 mol L^−1^ H_2_SO_4_	7.98	0.155	[43]
Nafion/GR/GCE	Soft drinks	DPV	0.01 mol L^−1^ H_2_SO_4_	0.12	0.00233	[22]
Nafion/MWCNTs/GCE	Beverage samples	DPV	0.01 mol L^−1^ H_2_SO_4_	0.23	0.00447	[44]
Nafion/BDDE	Real cola samples	DPV	0.2 mol L^−1^ H_2_SO_4_	0.10	0.00194	[14]
Nafion^®^/GCE	Energy drinks	DPV	0.1 mol L^−1^ Britton–Robinson buffer (pH 4.5)	18.9	0.367	[45]
Nafion/PST/GCE	Tea	LSD	H_2_SO_4_ solution (pH 1.0)	0.10	0.00194	[46]
CTAB/GR/GCE	Soft drink sample	DPV	0.01 mol L^−1^ H_2_SO_4_	0.091	0.00177	[47]
PAHNSA/GCE	Coffee extracts	SWV	Acetate buffer solution (pH 5)	0.137	0.00266	[48]
Pt/CNTs/GCE	Chinese tea and Cola beverage	DPV	0.01 mol L^−1^ H_2_SO_4_	0.20	0.00388	[20]
×GnP-ZrO_2_ nanocomposite-modified GCE	Various beverage	DPV	0.1 mol L^−1^ H_2_SO_4_	0.0119	0.00231	[49]
Modified MoS_2_/PANI@g-C_3_N_4_ electrode (GCE)	Red Bull energy drink	DPV	1 mmol L^−1^ Phosphate buffer solutions (PBS)	0.062	0.0012	[50]
MIP/CPE	Spiked beverage and tea samples	DPV	Phosphate buffer (pH 7)	0.015	0.000291	[51]
^3^ BQMCPE	Coffee	SWV	Phosphate buffer (pH 6)	0.30	0.00583	[52]
SWCNT/CPE	Coffee, tea, and cola nuts	DPV	0.01 mol L^−1^ H_2_SO_4_ pH 1.7	0.12	0.00233	[20]
^4^ Nitrogen-doped carbon/GCE	Green tea and energy drink sample	DPV	0.01 mol L^−1^ H_2_SO_4_–Na_2_SO_4_ (pH 1.70)	0.02	0.000388	[53]
Nitrogen-doped grafhene (NGR)	Cookie samples, chocolate and two kinds of milk tea	SWV	0.01 mol L^−1^ H_2_SO_4_	0.02	0.000388	[53]
^5^ Poly (ARS)	Energy drink	SWV	Acetate buffer	0.06	0.00117	[54]
GrRAC	Soft beverages	DPV	0.1 mol L^−1^ H_2_SO_4_	2.94	0.0571	[10]
GrRGC	Soft beverages	DPV	0.1 mol L^−1^ H_2_SO_4_	6.05	0.117	[11]
CF-µE	Soft drinks	DPV	0.5 mol L^−1^ H_2_SO_4_	0.13	0.00252	This work

^1^ Carbon active with ZnFe-modified glass carbon; ^2^ copper sulfide nanoparticle-modified carbon paste electrode; ^3^ 1,4-benzoquinone-modified carbon paste electrode; ^4^ glassy carbon electrode (GCE) modified with nitrogen-doped carbon nanotubes; ^5^ Simultaneous determination of CAF and VAN.

**Table 2:** Caffeine contents, as reported in the soft drinks, free of or containing caffeine, as well as HPLC and microelectrode determinations.

Beverages	Labelled/mg ^a^	HPLC/mg	CF-µE/mg	Error ^b^ (%)	Error ^c^ (%)
Caffeine-free soft drink	0.00	- ^d^	0.01	5	0
Soft drink 1	32.0	35.1	33.2	−9.68	−3.75
Soft drink 2	32.0	34.5	32.9	−7.81	3.23
Diet Soft drink 3	46.0	45.5	45.8	1.09	0.43
Zero sugar Soft drink 4	68.0	69.2	68.2	−1.76	−0.29
Energy drink	80.0 ^e^	78.2	79.5	2.25	0.63

^a^ Commercial coke soft drinks contain 32 mg of caffeine per 12-oz. (335 mL) serving. ^b^ Relative error (%) = [(Labelled value − voltammetric value)/(Labelled value) × 100]. ^c^ Relative error (%) = [(Labelled value − HPLC value)/(Labelled value) × 100]. ^d^ Under limit of instrumental detection. ^e^ Commercial energy drinks contain 80 mg of caffeine per 8.4-oz. (250 mL) serving.

## Data Availability

Not applicable.

## References

[1] Švorc L., Tomčík P., Svítková J., Rievaj M., Bustin D. (2012). Voltammetric determination of caffeine in beverage samples on bare boron-doped diamond electrode. Food Chem..

[2] Araujo D., Brito C., de Oliveira S.D., Silva D., Martinez-Huitle C., Aragao C. (2014). Platinum sensor for quantifying caffeine in drug formulations. Curr. Pharm. Anal..

[3] Rostagno M.A., Manchón N., D’Arrigo M., Guillamón E., Villares A., García-Lafuente A., Ramos A., Martínez J.A. (2011). Fast and simultaneous determination of phenolic compounds and caffeine in teas, mate, instant coffee, soft drink and energetic drink by high-performance liquid chromatography using a fused-core column. Anal. Chim. Acta.

[4] Rajabi Khorrami A., Rashidpur A. (2012). Development of a fiber coating based on molecular sol-gel imprinting technology for selective solid-phase micro extraction of caffeine from human serum and determination by gas chromatography/mass spectrometry. Anal. Chim. Acta.

[5] Rahim A.A., Nofrizal S., Saad B. (2014). Rapid tea catechins and caffeine determination by HPLC using microwave-assisted extraction and silica monolithic column. Food Chem..

[6] Al-Othman Z.A., Aqel A., Alharbi M.K.E., Yacine Badjah-Hadj-Ahmed A., Al-Warthan A.A. (2012). Fast chromatographic determination of caffeine in food using a capillary hexyl methacrylate monolithic column. Food Chem..

[7] Hadad G.M., Abdel Salam R.A., Soliman R.M., Mesbah M.K. (2012). Rapid and simultaneous determination of antioxidant markers and caffeine in commercial teas and dietary supplements by HPLC-DAD. Talanta.

[8] Svorc L. (2013). Determination of caffeine: A comprehensive review on electrochemical methods. Int. J. Electrochem. Sci..

[9] Vasilescu I., Eremia S.A.V., Penu R., Albu C., Radoi A., Litescu S.C., Radu G.L. (2015). Disposable dual sensor array for simultaneous determination of chlorogenic acid and caffeine from coffee. RSC Adv..

[10] Monteiro M.K.S., da Silva D.R., Quiroz M.A., Vilar V.J.P., Martínez-Huitle C.A., dos Santos E.V. (2021). Applicability of cork as novel modifiers to develop electrochemical sensor for caffeine determination. Materials.

[11] Monteiro M.K.S., Paiva S.S.M., da Silva D.R., Vilar V.J.P., Martínez-Huitle C.A., dos Santos E.V. (2019). Novel cork-graphite electrochemical sensor for voltammetric determination of caffeine. J. Electroanal. Chem..

[12] de Araújo D.M., Paiva S.d.S.S.M., Henrique J.M.M., Martínez-Huitle C.A., dos Santos E.V. (2021). Green composite sensor for monitoring hydroxychloroquine in different water matrix. Materials.

[13] Ören T., Anık Ü. (2017). Voltammetric determination of caffeine by using gold nanoparticle-glassy carbon paste composite electrode. Measurement.

[14] Martínez-Huitle C.A., Suely Fernandes N., Ferro S., de Battisti A., Quiroz M.A. (2010). Fabrication and application of Nafion®-modified boron-doped diamond electrode as sensor for detecting caffeine. Diam. Relat. Mater..

[15] Rick Lopes Da Silva Á., Medeiros De Araújo D., Bernardo Sabino Da Silva E., Serradella Vieira D., de Kássio Vieira Monteiro N., Martínez-Huitle C.A. (2017). Understanding the behavior of caffeine on a boron-doped diamond surface: Voltammetric, DFT, QTAIM and ELF Studies. New J. Chem..

[16] Yardim Y., Keskin E., Şentürk Z. (2013). Voltammetric determination of mixtures of caffeine and chlorogenic acid in beverage samples using a boron-doped diamond electrode. Talanta.

[17] Wong A., Santos A.M., Silva T.A., Fatibello-Filho O. (2018). Simultaneous determination of isoproterenol, acetaminophen, folic acid, propranolol and caffeine using a sensor platform based on carbon black, graphene oxide, copper nanoparticles and PEDOT:PSS. Talanta.

[18] Mahanthappa M., Yellappa S., Kottam N., Srinivasa Rao Vusa C. (2016). Sensitive determination of caffeine by copper sulphide nanoparticles modified carbon paste electrode. Sens. Actuators A Phys..

[19] Wang Y., Ding Y., Li L., Hu P. (2018). Nitrogen-doped carbon nanotubes decorated Poly (L-Cysteine) as a novel, ultrasensitive electrochemical sensor for simultaneous determination of theophylline and caffeine. Talanta.

[20] Habibi B., Abazari M., Pournaghi-Azar M.H. (2012). A carbon nanotube modified electrode for determination of caffeine by differential pulse voltammetry. Chin. J. Catal..

[21] AL-Gahouari T., Bodkhe G., Sayyad P., Ingle N., Mahadik M., Shirsat S.M., Deshmukh M., Musahwar N., Shirsat M. (2020). Electrochemical Sensor: L-Cysteine induced selectivity enhancement of electrochemically reduced graphene oxide–multiwalled carbon nanotubes hybrid for detection of lead (Pb*^2^*^+^) ions. Front. Mater..

[22] Sun J.Y., Huang K.J., Wei S.Y., Wu Z.W., Ren F.P. (2011). A graphene-based electrochemical sensor for sensitive determination of caffeine. Colloids Surf. B Biointerfaces.

[23] Jeevagan A.J., John S.A. (2012). Electrochemical determination of caffeine in the presence of paracetamol using a self-assembled monolayer of non-peripheral amine substituted copper(II) phthalocyanine. Electrochim. Acta.

[24] Ghoreishi S.M., Attaran A.M., Amin A.M., Khoobi A. (2015). Multiwall carbon nanotube-modified electrode as a nanosensor for electrochemical studies and stripping voltammetric determination of an antimalarial drug. RSC Adv..

[25] Rassaei L., Marken F., Sillanpää M., Amiri M., Cirtiu C.M., Sillanpää M. (2011). Nanoparticles in electrochemical sensors for environmental monitoring. Trends Anal. Chem..

[26] Nebel C.E., Yang N., Uetsuka H., Osawa E., Tokuda N., Williams O. (2009). Diamond nano-wires, a new approach towards next generation electrochemical gene sensor platforms. Diam. Relat. Mater..

[27] Carolina Torres A., Barsan M.M., Brett C.M.A. (2014). Simple electrochemical sensor for caffeine based on carbon and nafion-modified carbon electrodes. Food. Chem..

[28] Siqueira G.P., de Faria L.V., Rocha R.G., Matias T.A., Richter E.M., Muñoz R.A.A., da Silva I.S., Dantas L.M.F. (2022). Nanoporous gold microelectrode arrays using microchips: A highly sensitive and cost-effective platform for electroanalytical applications. J. Electroanal. Chem..

[29] Jose J., Subramanian V., Shaji S., Sreeja P.B. (2021). An electrochemical sensor for nanomolar detection of caffeine based on Nicotinic Acid Hydrazide Anchored on Graphene Oxide (NAHGO). Sci. Rep..

[30] Sachidananda T.G., Chikkanagoudar R.N., Pattar N., Nandurkar S. (2022). Investigations of the influence of geometrical parameters of carbon nanotube material for sensor and MEMS applications. Mater. Today Proc..

[31] Mao S., Lu G., Chen J. (2014). Nanocarbon-based gas sensors: Progress and challenges. J. Mater. Chem. A Mater..

[32] Wang B., Wen X., Chiou P.-Y., Maidment N.T. (2019). Pt nanoparticle-modified carbon fiber microelectrode for selective electrochemical sensing. Electroanalysis.

[33] Aquino de Queiroz J.L., Martínez-Huitle C.A., Castro P.S. (2020). Real time monitoring of in situ generated hydrogen peroxide in electrochemical advanced oxidation reactors using an integrated Pt microelectrode. Talanta.

[34] Brunetti B., Desimoni E., Casati P. (2007). Determination of caffeine at a nation-covered glassy carbon electrode. Electroanalysis.

[35] Arroyo-Gómez J.J., Villarroel-Rocha D., de Freitas-Araújo K.C., Martínez-Huitle C.A., Sapag K. (2018). Applicability of activated carbon obtained from peach stone as an electrochemical sensor for detecting caffeine. J. Electroanal. Chem..

[36] Khoo W.Y.H., Pumera M., Bonanni A. (2013). Graphene platforms for the detection of caffeine in real samples. Anal. Chim. Acta.

[37] Currie L.A. (1995). International Union of Pure and Applied Chemistry Nomenclature in Evaluation of Analytical Methods Including Detection and Quantification Capabilities. Pure Appl. Chem..

[38] Desimoni E., Brunetti B. (2009). About estimating the limit of detection of heteroscedastic analytical systems. Anal. Chim. Acta.

[39] Danzer K., Currie L.A. (1998). Guideline for calibration in analytical chemistry—Part 1. Fundamentals and single component calibration. Pure Appl. Chem..

[40] Redivo L., Stredanský M., DeAngelis E., Navarini L., Resmini M., Švorc Ĺ. (2018). Bare carbon electrodes as simple and efficient sensors for the quantification of caffeine in commercial beverages. R. Soc. Open Sci..

[41] Lourenção B.C., Antigo Medeiros R., Rocha-Filho R.C., Mazo L.H., Fatibello-Filho O. (2009). Simultaneous voltammetric determination of paracetamol and caffeine in pharmaceutical formulations using a boron-doped diamond electrode. Talanta.

[42] Jesu Amalraj A.J., Umesh N., Wang S.F. (2022). Rational design of platinum assimilated 3-D zinc cobalt oxide flowers for the electrochemical detection of caffeine in beverage and energy drink. J. Ind. Eng. Chem..

[43] Tyszczuk-Rotko K., Bęczkowska I. (2015). Nafion covered lead film electrode for the voltammetric determination of caffeine in beverage samples and pharmaceutical formulations. Food Chem..

[44] Yang S., Yang R., Li G., Qu L., Li J., Yu L. (2010). Nafion/Multi-wall carbon nanotubes composite film coated glassy carbon electrode for sensitive determination of caffeine. J. Electroanal. Chem..

[45] Farag A.S., Pravcová K., Česlová L., Vytřas K., Sýs M. (2019). Simultaneous determination of caffeine and pyridoxine in energy drinks using differential pulse voltammetry at glassy carbon electrode modified with Nafion®. Electroanalysis.

[46] Guo S., Zhu Q., Yang B., Wang J., Ye B. (2011). Determination of caffeine content in tea based on Poly(Safranine T) electroactive film modified electrode. Food Chem..

[47] Sun J.Y., Huang K.J., Wei S.Y., Wu Z.W. (2011). Application of cetyltrimethylammonium bromide–graphene modified electrode for sensitive determination of caffeine. Can. J. Chem..

[48] Amare M., Admassie S. (2012). Polymer modified glassy carbon electrode for the electrochemical determination of caffeine in coffee. Talanta.

[49] Okutan M., Boran F., Alver E., Asan A. (2022). One-pot synthesize of graphene-ZrO_2_ nanocomposite: A novel modified glassy carbon electrode for the detection of caffeine in beverage samples. Mater. Chem. Phys..

[50] Murugan E., Dhamodharan A. (2021). Separate and simultaneous determination of vanillin, theophylline and caffeine using molybdenum disulfide embedded polyaniline/graphitic carbon nitrite nanocomposite modified glassy carbon electrode. Diam. Relat. Mater..

[51] Alizadeh T., Ganjali M.R., Zare M., Norouzi P. (2010). Development of a voltammetric sensor based on a Molecularly Imprinted Polymer (MIP) for caffeine measurement. Electrochim. Acta.

[52] Aklilu M., Tessema M., Redi-Abshiro M. (2008). Indirect voltammetric determination of caffeine content in coffee using 1,4-Benzoquinone modified carbon paste electrode. Talanta.

[53] Jiang L., Ding Y., Jiang F., Li L., Mo F. (2014). Electrodeposited nitrogen-doped graphene/carbon nanotubes nanocomposite as enhancer for simultaneous and sensitive voltammetric determination of caffeine and vanillin. Anal. Chim. Acta.

[54] Filik H., Avan A.A., Mümin Y. (2017). Simultaneous electrochemical determination of caffeine and vanillin by using Poly(Alizarin Red S) modified glassy carbon electrode. Food Anal. Methods.

[55] Tadesse Y., Tadese A., Saini R.C., Pal R. (2013). Cyclic voltammetric investigation of caffeine at anthraquinone modified carbon paste electrode. Int. J. Electrochem..

